# D-serine metabolism enhances *Escherichia coli* fitness in the gut and could contribute to *Enterobacteriaceae* expansion in Crohn's disease patients

**DOI:** 10.1016/j.crmicr.2026.100585

**Published:** 2026-03-23

**Authors:** Maria Ines Moreira de Gouveia, Amelie Blanchot, Annie Garrivier, Julien Daniel, Anvi Laetitia Nguyen, Aurélie Messager, Annick Bernalier-Donadille, Gregory Jubelin

**Affiliations:** a Université Clermont Auvergne, INRAE, MEDIS UMR454, F-63000 Clermont-Ferrand, France; b Université Paris-Saclay, CEA, INRAE, Département Médicaments et Technologies pour La Santé (DMTS), MetaboHUB, F-91191, Gif sur Yvette, France

**Keywords:** Gut dysbiosis, Crohn’s disease, Rheumatoid arthritis, *Enterobacteriaceae*, Nutritional competition, D‑serine

## Abstract

Gut dysbiosis associated with several intestinal and extra-intestinal diseases is frequently characterized by an expansion of *Enterobacteriaceae*, which typically represent only a few percent of the total gut microbiota in healthy individuals. This expansion can be partially explained by the selection of strains that are metabolically adapted to the altered gut environment. In this study, we performed a phenotypic screening to identify substrates that are differentially catabolized by commensal *Escherichia coli* strains isolated from healthy individuals (HI) or from patients with Crohn’s disease (CD) or rheumatoid arthritis (RA), two inflammatory diseases associated with gut dysbiosis. While RA strains were metabolically similar to HI strains, CD strains displayed a metabolic pattern with greater dissimilarities. Among the substrates identified, we notably observed opposite metabolic capacities of HI and CD strains to utilize D‑serine and sucrose, a phenotype arising from genetic rearrangements within the argW locus of the *E. coli* chromosome. The D‑serine–positive phenotype of CD strains was shown to depend on the presence of the dsdCXA genes and to confer a nutritional advantage both *in vitro* and *in vivo*. Overall, our findings provide new insights into the metabolic traits that could drive the expansion of *Enterobacteriaceae* under dysbiotic conditions and highlight D‑serine utilization as a key determinant of *E. coli* fitness in the gut.

## Highlights

• *Escherichia coli* isolates from healthy or diseased hosts exhibit distinct metabolic profiles.
• D‑serine utilization is more prevalent in *E. coli* isolates from Crohn’s patients.
• The D‑serine associated ***dsdCXA*** cluster enhances *E. coli* fitness in the gut.

## Graphical abstract

**Figure:**
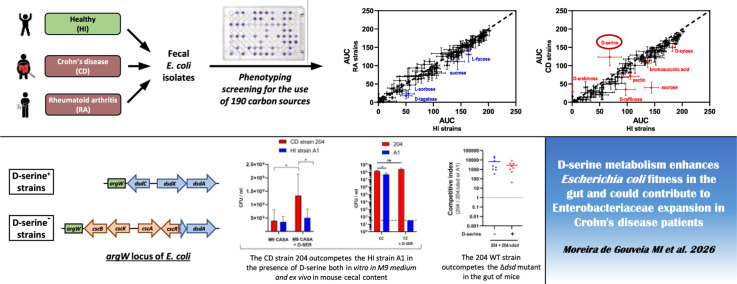

## Introduction

Throughout adult life, the gut microbiota of healthy individuals (HI) is considered relatively stable and plays a fundamental role in human health. Under homeostatic conditions, the gut microbiota contributes to host energy metabolism and fulfills essential structural, protective, and immunological functions (Clemente et al., 2012). Disruption of this equilibrium, commonly referred to as gut dysbiosis, can have detrimental effects on host health. Dysbiosis is typically characterized by features such as reduced microbial diversity and richness, a decrease in beneficial microbial taxa, and/or an increase in potentially harmful bacteria, notably members of the Proteobacteria phylum (Carding et al., 2015). In healthy individuals, Proteobacteria usually represent only 1–5% of the gut microbiota. However, their relative abundance can markedly increase during dysbiosis associated with a wide range of intestinal and extra-intestinal diseases (Degruttola et al., 2016). Within this phylum, the *Enterobacteriaceae* family, and particularly *E. coli*, plays a prominent role in the expansion of Proteobacteria observed under pathological conditions. Indeed, enrichment of Proteobacteria in the gut has been proposed as a marker of microbial instability and as a potential indicator of disease-associated dysbiosis (Rizzatti et al., 2017; Shin et al., 2015; Winter and Bäumler, 2014). Several non-mutually exclusive hypotheses have been proposed to explain the expansion of Proteobacteria in a dysbiotic gut environment (Moreira de Gouveia et al., 2024). One major mechanism involves disruption of physiological hypoxia within the intestinal lumen. Inflammation, epithelial dysfunction, or antibiotic treatment can increase oxygen diffusion into the gut lumen, thereby favoring the growth of facultative anaerobes such as *Enterobacteriaceae* (Rivera-Chávez et al., 2017). In addition, elevated levels of host-derived molecules that can serve as alternative electron acceptors for anaerobic respiration may further promote Proteobacteria expansion, as respiratory metabolism yields more energy than fermentation, which predominates in other members of the gut microbiota (Winter and Bäumler, 2023). Another hypothesis centers on the selection or adaptation of bacterial strains that are particularly well suited to the altered gut environment associated with disease. For example, patients with cystic fibrosis exhibit impaired absorption of proteins and lipids, leading to increased concentrations of glycerol in the gut lumen (Wouthuyzen-Bakker et al., 2011). Associated dysbiosis is characterized by a bloom of *E. coli* strains which grow faster than other commensal *E. coli* strains in presence of glycerol. Consistent with this notion of adaptability, *Enterobacteriaceae* display a high degree of genome plasticity and represent a major source of variable genes within the human gut microbiome, despite their relatively low abundance in healthy individuals (Bradley and Pollard, 2017). This genetic variability confers substantial metabolic flexibility, enabling rapid adaptation to changes in the gut environment.

The aim of our study was to explore this hypothesis in the context of two inflammatory diseases associated with Proteobacteria expansion: rheumatoid arthritis (RA) and Crohn’s disease (CD) (M. Baumgart et al., 2007; Chiang et al., 2019; Khorsand et al., 2022; Wang et al., 2022; Wu et al., 2016). RA is a chronic autoimmune inflammatory disease primarily affecting the joints and characterized by persistent systemic immune activation (Harris, 1990). CD is a chronic, segmental inflammatory bowel disease that can affect any region of the gastrointestinal tract, from the mouth to the anus (D. C. Baumgart and Sandborn, 2007). CD-associated dysbiosis is notably marked by a high prevalence of a specific pathotype known as adherent-invasive *E. coli* (AIEC), which contributes to disease pathogenesis (Chervy et al., 2020). Here, we performed a comparative phenotypic screening of *E. coli* strains isolated from fecal samples of healthy individuals and from patients with RA or CD to assess their capacity to metabolize a broad panel of carbon substrates. Several carbohydrates and amino acids were differentially consumed among the strain groups. Notably, the ability to catabolize D‑serine and sucrose differed markedly between HI and CD strains. These two metabolic traits are determined by distinct gene clusters that are both located within the highly variable *argW* locus of the *E. coli* genome (Jahreis et al., 2002). The D‑serine–positive phenotype observed in CD strains was associated with the presence of the *dsdCXA* genes and conferred a competitive growth advantage over HI strains under different conditions.

## Materials and methods

### Bacterial strains and growth conditions

*Enterobacteriaceae* strains were isolated from fecal samples obtained from healthy individuals (HI), patients with rheumatoid arthritis (RA), or patients with Crohn’s disease (CD). All participants gave their written informed consent and the study was approved by the local Human Ethics Committee (CPP Sud Est VI, France). The strains used in this study are listed in Table S1. For HI and RA individuals, stool samples were diluted and plated on MacConkey and Hektoen agar. Several colonies from each sample were subjected to RAPD-PCR analysis (Nielsen et al., 2014) in order to identify and exclude clonal isolates from the collection. Taxonomic identification was performed by amplification and sequencing of the 16S rDNA gene, and only Escherichia strains were retained. CD-associated strains were kindly provided by the M2iSH laboratory (Clermont-Ferrand, France). The phylogroup of each isolate was determined as desbrided in Clermont *et al*. (Clermont et al., 2013) or using the website http://clermontyping.iame-research.center/. *E. coli* strains were routinely cultured in LB medium or in a modified M9 minimal salts medium containing Na₂HPO₄ (42 mM), KH₂PO₄ (22 mM), NaCl (8.5 mM), MgSO₄ (2 mM), and CaCl₂ (0.1 mM). Carbon and/or nitrogen sources added to M9 medium were glucose (0.2%), casamino acids (CASA; 0.2%), D‑serine (0.2%), or NH₄Cl (18.5 mM). For growth experiments in M9 medium, strains were first streaked onto LB agar plates, and precultures were prepared by inoculating a single colony into LB medium and incubating for 8 h at 37 °C with shaking. Precultures were then diluted 1:100 into M9 medium supplemented with glucose and grown overnight. Cells were harvested, washed once with M9 medium, and used to inoculate experimental cultures containing the indicated carbon sources. Cultures were incubated at 37 °C with shaking, and growth was monitored by measuring optical density at 600 nm (OD₆₀₀). When required, antibiotics were used at the following final concentrations: kanamycin (25 µg·mL⁻¹), gentamicin (15 µg·mL⁻¹), and ampicillin (100 µg·mL⁻¹).

### Construction of strains

A gentamicin-resistant (Gmᴿ) derivative of strain 204 was generated by insertion of a gentamicin resistance cassette at the chromosomal attTn7 site, as previously described (Crepin et al., 2012). Correct insertion was verified by PCR, and the resulting strain (204-Gm) exhibited growth kinetics similar to those of the wild-type strain. Deletion of the *dsd* locus in strain 204 was achieved using the one-step PCR-based gene inactivation method (Datsenko and Wanner, 2000), replacing the locus with a kanamycin resistance cassette. Successful deletion was confirmed by PCR amplification and sequencing using primers specific for the *yfdC* and *emrY* genes flanking the *dsd* locus. All primers used for mutant construction and verification are listed in Table S2.

### Phenotype microarray experiments

Phenotype MicroArray analysis (Biolog®) was used as a semi–high-throughput approach to characterize microbial metabolic phenotypes. Plates PM01 and PM02 were employed to assess the utilization of 190 different carbon substrates. Strains were revived from −80 °C stocks on LB agar plates, and a single colony was re-streaked onto fresh LB plates one day prior to the experiment. For each strain, multiple colonies were collected using a cotton swab and resuspended in IF-0 1X inoculating fluid supplemented with Dye A until a transmittance of 85% was reached, as measured with a Biolog® turbidimeter. Each well of the PM plates was inoculated with 100 µL of the bacterial suspension, and plates were incubated in the OmniLog system at 37 °C for 24 h. Colorimetric readings were recorded every 15 min. Raw kinetic data were exported using the OmniLog Data Analysis Software (version 1.7), and the area under the curve (AUC) values were calculated and used for subsequent analyses.

### Selection of strains representative of HI, CD and RA groups

Representative strains for each group were selected using a scoring approach. First, for each substrate, the percentage of strains able to metabolize it was calculated within each group. A substrate was considered to be utilized by a group when >66% of strains displayed a positive phenotype. Conversely, if fewer than 33% of strains were able to metabolize a substrate, the group was considered unable to use it. Substrates with intermediate utilization frequencies (33–66%) were considered heterogeneous and excluded from the analysis. For each strain, a score was calculated by assigning one point for each substrate for which the strain’s phenotype matched the consensus phenotype of its group. Strains with the highest scores were considered the most representative of their respective groups.

### Detection of dsd and csc operons

The presence of the *dsd* and *csc* operons was assessed by triplex PCR using genomic DNA extracted from the indicated strains and primers *dsdA_R, cscR_F*, and *dsdX_F* (Table S2). PCR amplification was performed using Taq DNA polymerase under the following conditions: initial denaturation at 94 °C for 2 min; 35 cycles of denaturation at 94 °C for 1 min, annealing at 57 °C for 30 s, and extension at 72 °C for 1 min; followed by a final extension at 72 °C for 5 min. PCR products were separated by agarose gel electrophoresis and visualized after ethidium bromide staining. For sequenced strains, in silico triplex PCR analyses were performed using the same primer sequences, and results were visualized as virtual agarose gels using SnapGene® software (Insightful Science).

### **D**- and L‑serine quantification

For amino acid quantification, 50 µL of each sample was precipitated by adding 150 µL of methanol. Subsequently, 5 µL of L‑serine-¹³C₃¹⁵N (used as an internal standard for both L- and D‑serine) was added to a final concentration of 9.73 µg·mL⁻¹. Samples were centrifuged at 20,000 × *g* for 10 min at 4 °C, and 10 µL of the supernatant was diluted in 75 µL of water containing 0.3% trifluoroacetic acid (TFA; Merck, Saint-Quentin-Fallavier, France). Analyses were performed using ultra-performance liquid chromatography coupled to tandem mass spectrometry (UPLC-MS/MS) on an ACQUITY UPLC® I-Class system connected to a Xevo™ TQ-XS triple quadrupole mass spectrometer (Waters, Guyancourt, France). Separation was achieved using a Crownpak CR-I(+) column (5 µm, 3.0 × 150 mm; Daicel, Illkirch, France) maintained in an ice bath. Mobile phase A consisted of water with 0.3% TFA, and mobile phase B consisted of acetonitrile with 0.3% TFA. Following injection of 5 µL of sample, isocratic elution was performed using a 90:10 (v/v) water/acetonitrile mixture containing 0.3% TFA for 10 min. The column effluent was introduced directly into the electrospray ionization (ESI) source operating in positive ion mode with multiple reaction monitoring (MRM). The monitored transitions were *m/z* 106 → 60 for both D- and L‑serine, and *m/z* 113 → 66 for the internal standard. Mean retention times were approximately 4.55 min for D‑serine and 4.88 min for L‑serine. Data acquisition and analysis were performed using MassLynx software (version 4.2). Quantification was achieved using calibration curves generated from peak area ratios relative to the internal standard, applying linear regression with 1/x weighting.

### *In vitro* competition experiments

Competition assays between strain 204-Gm and strain A1 (naturally resistant to ampicillin) were conducted in M9 medium supplemented with either 0.2% casamino acids alone or a mixture of 0.2% casamino acids and 0.2% D‑serine. Strains were grown overnight in LB medium, washed once with M9 medium, and used to inoculate experimental cultures at a 1:1 ratio (10⁷ cells of each strain per mL). Cultures were incubated at 37 °C with shaking for 8 h, and OD₆₀₀ was recorded hourly. After 8 h, samples were diluted in PBS and plated on LB agar supplemented with gentamicin or ampicillin to enumerate strains 204-Gm and A1, respectively.

### Mouse experiments

Specific-pathogen-free (SPF) C57BL/6 mice were purchased from Charles River Laboratories. Five- to six-week-old mice were housed in groups of no more than five per cage, maintained under a 12 h light/dark cycle at 21 ± 2 °C, and provided with standard chow and water *ad libitum*. All experiments were reviewed and approved by the Auvergne Committee for Animal Experimentation (C2EA) under agreement number #476852024021511228246 v3. For *ex vivo* experiments, bacterial growth was assessed in a medium prepared from mouse cecal contents. Cecal contents from four mice were pooled and diluted in sterile PBS (20 mg per mL). Samples were centrifuged for 5 min at 500 × *g*, and the supernatant was transferred and centrifuged again for 5 min at 10,000 × *g*. The resulting supernatant was filtered through a 0.22 µm membrane, aliquoted, and stored at −80 °C. Sterility was confirmed by plating on LB agar and incubating for 3 days at 37 °C. For competition assays, strains 204-Gm and A1 were grown overnight in LB medium, washed once with PBS, and inoculated into cecal content medium at a 1:1 ratio (10³ cells of each strain per mL). Cultures were incubated at 37 °C under 5% CO₂ for 8 h, with OD₆₀₀ measured every 30 min. Bacterial counts were determined by plating on selective media.

For *in vivo* co-inoculation experiments, mice received drinking water supplemented with streptomycin sulfate (5 *g*·L⁻¹) from day −5 to day −2 (day 0 corresponding to bacterial inoculation) to deplete facultative anaerobic bacteria and open the ecological niche for *E. coli*. In some groups, drinking water was further supplemented with 0.2% D‑serine from day −1 to day 5. On day 0, mice were intragastrically co-inoculated with 100 µL of PBS containing 2 × 10⁸ CFU of bacterial mixtures (204-Gm/A1 or 204-Gm/204Δdsd at a 1:1 ratio). Fecal samples were collected at day 5 post-inoculation, diluted, and plated on LB agar supplemented with gentamicin, ampicillin, or kanamycin to enumerate the respective strains. Competitive indices were calculated as the ratio of output CFU divided by the input ratio.

## Statistical analysis

Statistical analyses were performed using GraphPad Prism software (version 8; San Diego, *CA*, USA). Outliers were identified and excluded using the ROUT method with a Q value of 1%. The statistical tests applied for each dataset are specified in the corresponding figure legends. * *P* < 0.05; ** *P* < 0.01; *** *P* < 0.001; ns: non-significant.

## Results

### Phenotype microarrays identified carbon substrates differentially consumed between HI and CD or RA strains

**Fig. 1:**
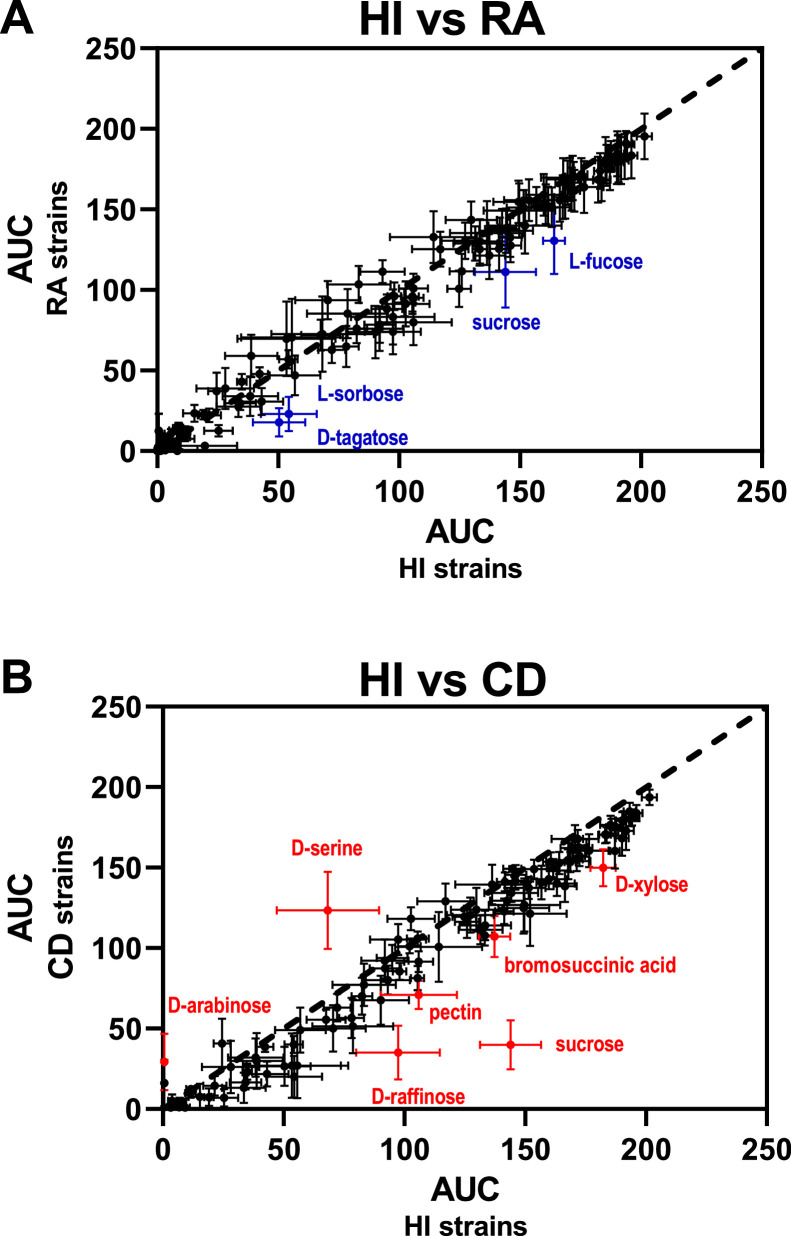
AUC comparison between HI strain group and CD or RA strain groups. The AUC calculated by the OmniLog Data Analysis Software were plotted on a 2-axis graph in which x-axis corresponds to AUC from HI strain group and y-axis correspond to RA (A) or CD (B) strain groups. Data are represented by black dots and associated error-bars (standard errors of the mean). A two-way ANOVA test with the Holm-Sidak's correction was performed to compare the 3 groups for each substrate. Significant differences (*P* < 0.01) are indicated with blue symbols in panel A (HI *vs* RA) or with red symbols in panel B (HI *vs* CD).

Phenotype MicroArray screening using the OmniLog system was performed on 16 HI strains, 14 RA strains, and 15 CD strains. Plates PM01 and PM02 were used to evaluate the utilization of 190 different substrates as sole carbon sources. To obtain a qualitative overview of potential metabolic differences between strain groups, the OmniLog Data Analysis Software generated overlay plots of mean growth curves comparing two groups at a time (Figure S1). Of note, *E. coli* strains were unable to utilize several substrates as sole carbon sources, particularly those included in PM02, which contains polymers, amino acids, and L-sugars. For most substrates, similar metabolic profiles were observed across groups. However, notable differences were detected between HI strains and strains isolated from RA or CD patients. To further investigate these differences, a quantitative analysis was performed by extracting the area under the curve (AUC) values for each substrate. AUC values were then compared between strain groups (Fig. 1). Only a limited number of statistically significant differences were observed between HI and RA strains. Specifically, *E. coli* strains isolated from RA patients displayed significantly lower AUC values for D-tagatose, L-sorbose, L-fucose, and sucrose compared with HI strains (*P* ≤ 0.01) (Fig. 1A). In contrast, a larger number of substrates were differentially consumed between HI and CD strains, including saccharides, amino acids, and carboxylic acids (Fig. 1B). Among these, the most pronounced differences were observed for D‑serine and sucrose, which were preferentially consumed by CD strains and HI strains, respectively.

### The ability to consume **D**-Serine and sucrose is opposite between HI and CD strains

**Fig. 2:**
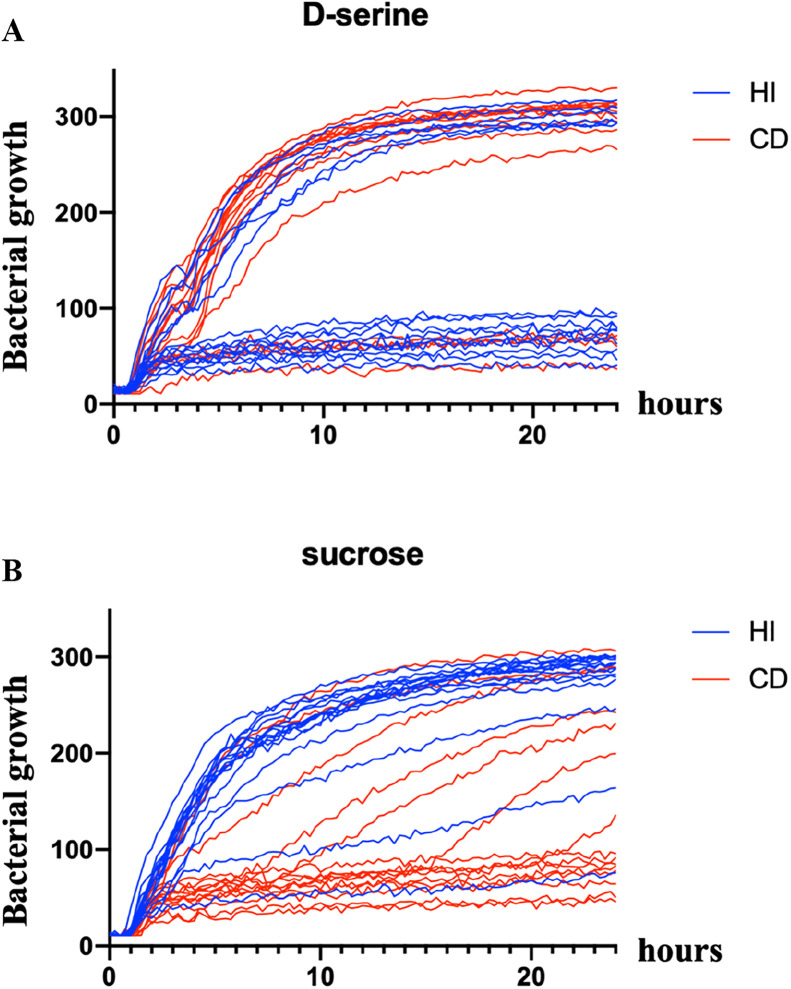
Representation of growth curves for individual HI and CD strains in presence of D-serine or sucrose as a carbon source. Growth curves are represented as a quantification of tetrazolium dye reduction (y) in function of time (x) for D-serine (A) and sucrose (B). Growth curves of HI strains are in blue and growth curves of CD strains are in red.

**Fig. 3:**
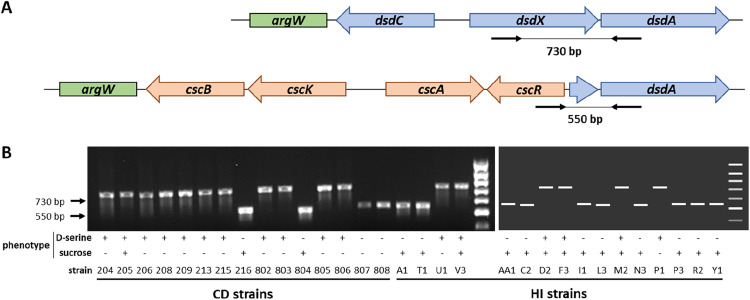
Correlation between D-serine and sucrose utilization phenotypes and presence of *dsd* and *csc* loci in HI and CD strains. (A) Schematic representations of the *argW* locus in *Escherichia coli*. Strains present either the *dsd* locus (blue arrows) responsible for the utilization of D-serine or the *csc* locus (orange arrows) responsible for sucrose utilization. A triplex PCR using primers (thick black arrows) annealing sequences in *dsdX, dsdA* or *cscR* genes was used to detect *argW* configuration in indicated strains. (B) Electrophoresis gel of the triplex PCR (left panel) and *in silico* gel for strains with available genome (right panel). A PCR fragment of 730 bp indicates the presence of the *dsd* locus and a PCR fragment of 550 bp indicates the presence of the *csc* locus. D-serine and sucrose utilization phenotypes are indicated with + or – symbols.

One of the most striking findings from the phenotype microarray analysis was the inverse correlation between D‑serine and sucrose utilization in HI and CD strain groups. The *dsdCXA* and *cscRAKB* gene clusters, which are involved in the catabolism of D‑serine and sucrose, respectively, are both located within the chromosomal *argW* locus in *E. coli*. This locus is known to be highly variable, with many strains harboring a replacement of the *dsdCXA* genes by the sucrose utilization genes *cscRAKB* (Jahreis et al., 2002; Moritz and Welch, 2006). We therefore focused our analysis on the metabolic capacity of HI and CD strains to utilize these two substrates. Individual growth curves from the OmniLog screening were examined for D‑serine and sucrose (Fig. 2). Only 37% of HI strains (6/16) were able to grow with D‑serine as the sole carbon source, whereas >73% of CD strains (11/15) exhibited this ability. Conversely, 94% of HI strains (15/16) were able to utilize sucrose, compared with only 40% of CD strains (6/15). To link these phenotypes to the genetic organization of the *argW* locus, we investigated the presence of the *dsd* and *csc* loci using a triplex PCR targeting *dsdX, dsdA*, and *cscR*. Amplification of a 730 bp fragment indicated the presence of the *dsd* locus, whereas a 550 bp fragment indicated the presence of the *csc* locus (Fig. 3A). As shown in Fig. 3B, the ability to metabolize D‑serine was consistently associated with the presence of the *dsd* locus in both HI and CD strains. Similarly, with the exception of two strains, sucrose utilization correlated with detection of the *csc* locus. Notably, a subset of strains did not display a strictly inverse phenotype and were either able or unable to metabolize both substrates. This observation could be explained, at least partially, by the existence of alternative sucrose utilization pathways in *E. coli* (Stephens et al., 2024). Overall, these results indicate that the capacity to catabolize D‑serine is significantly more prevalent among CD strains than among HI strains, a phenotype that can be directly attributed to the presence of the *dsd* genes within the *argW* locus.

### Utilization of **D**‑serine confers a growth advantage to CD strains over HI strains *in vitro*

**Fig. 4:**
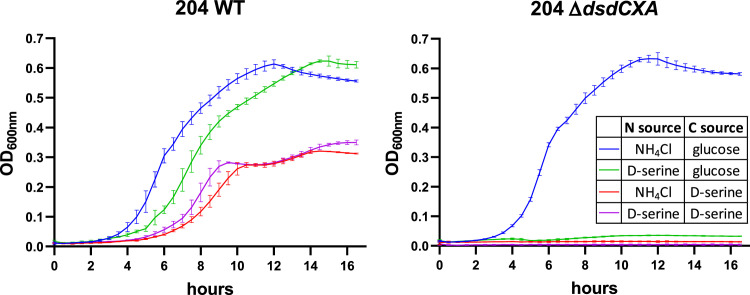
Growth of the WT and ∆*dsdCXA* mutant 204 strains. Growth was performed in M9 minimal media supplemented with indicated nitrogen and carbon sources. The optical density at 600 nm (OD_600nm_) was measured every 30 min for 17 h. Growth curves are presented as the mean and standard deviation from 3 independent replicates.

**Fig. 5:**
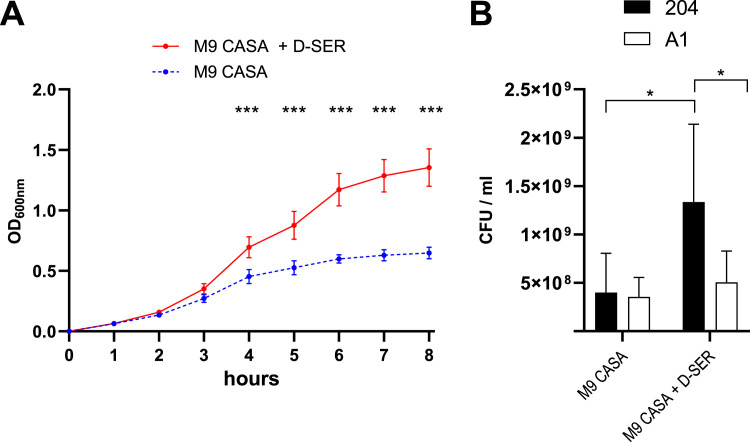
*In vitro* competition assays between HI strain A1 and CD strain 204. Competitions were carried out in M9 + casamino acids (CASA) 0.2% and supplemented or not with D-serine 0.2%. Growth curves (A) and numeration of each strain at 8 h (B) are presented as the mean and standard deviation from 5 independent replicates. Multiple t tests with Holm-Sidak's correction were performed to compare OD_600_ values and a two-way ANOVA with Holm-Sidak's correction was performed to compare bacterial concentration.

To assess the impact of D‑serine utilization on *E. coli* fitness, representative strains from the HI and CD groups were selected. This was achieved by comparing the metabolic phenotype of individual strains to the average phenotype of their respective group (see Materials and Methods for details). Strains A1 (HI) and 204 (CD) were identified as the most representative strains (Figure S2). As expected, strain A1 exhibited a sucrose⁺/D‑serine⁻ phenotype, whereas strain 204 displayed a sucrose⁻/D‑serine⁺ phenotype. To confirm that D‑serine utilization by strain 204 depended on the *dsd* cluster, a Δ*dsdCXA* mutant was constructed and its growth was assessed in minimal medium supplemented with D‑serine as the sole carbon and/or nitrogen source. While the wild-type strain grew efficiently under these conditions, the Δ*dsdCXA* mutant was unable to grow (Fig. 4). Quantification of D‑serine confirmed its consumption by strain 204 during growth (Figure S3), demonstrating that D‑serine catabolism is mediated by a functional *dsd* locus. We then evaluated whether D‑serine provides a competitive advantage to CD strains over HI strains by performing *in vitro* competition assays between strains 204 and A1 in minimal medium containing casamino acids as a nutrient source and supplemented or not with D‑serine (Fig. 5). In the absence of D‑serine supplementation, both strains grew similarly, and the low amount of D‑serine present in casamino acids was fully consumed (Figs. 5 and S3). In contrast, supplementation with D‑serine increased the maximal OD_600nm_ from approximately 0.6 to 1.3, a difference attributable to the overgrowth of strain 204, which efficiently utilized D‑serine.

### **D**‑serine exerts a deleterious effect on dsd^-^ strains in an intestinal environment

**Fig. 6:**
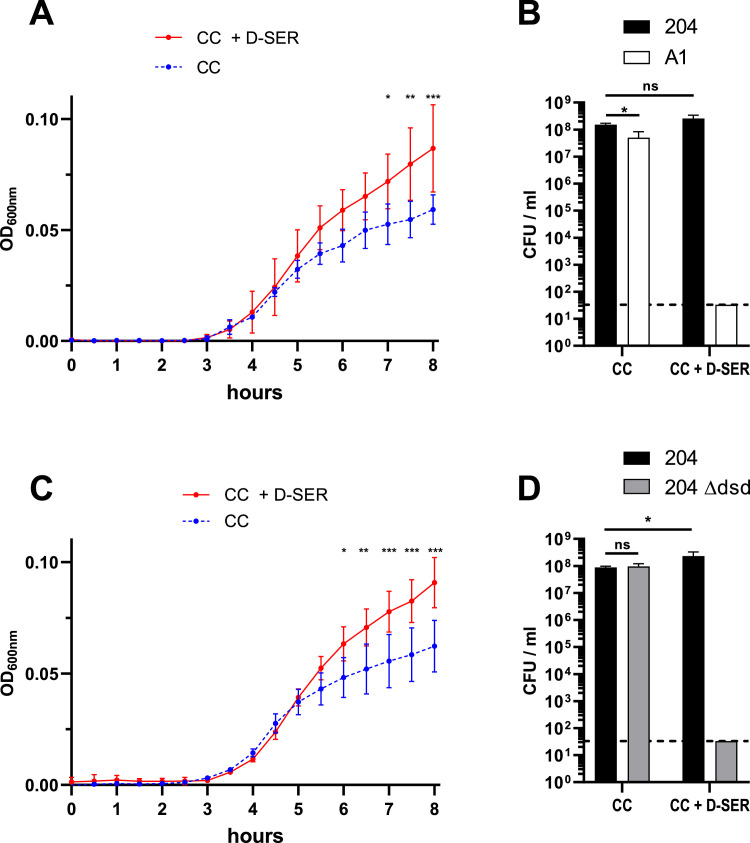
*Ex vivo* competition in mouse cecal content. Co-cultures of 204 + A1 (A and B) or 204 + 204 ∆*dsd* (C and D) were performed in cecal content (CC) of mouse supplemented or not with D-serine 0.2%. Growth curves and numeration of each strain at 8 h are presented as the mean and standard deviation from 3 independent replicates. The dashed lines in panel C and D represent the limit detection of bacterial concentration in samples. Multiple t tests with Holm-Sidak's correction were performed to compare OD_600_ values and a two-way ANOVA with Holm-Sidak's correction was performed to compare bacterial concentration.

**Fig. 7:**
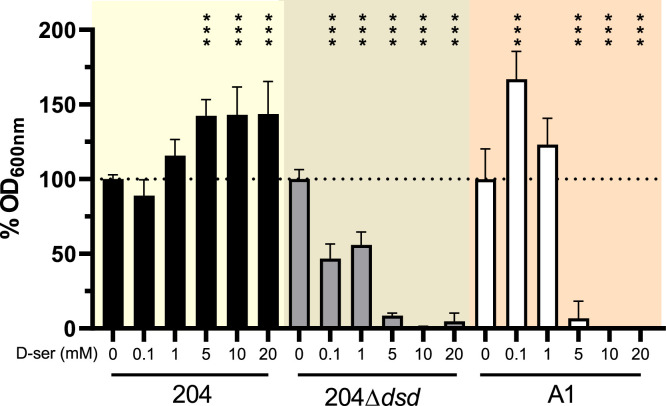
*Toxic effect of D-serine in absence of the dsd operon*. Growth was performed in mouse cecal content supplemented with indicated concentrations of D-serine. OD_600nm_ was measured at 8 h and values are shown as percentages relative to OD_600nm_ measured without D-serine supplementation for each strain. Data represent the mean and standard deviation from 3 independent replicates. A two-way ANOVA test with the Holm-Sidak's correction was performed to compare values with the control condition of each strain (no D-serine supplementation).

To further investigate the role of D‑serine in an intestinal context, we evaluated bacterial growth under *ex vivo* conditions using diluted mouse cecal content (CC). When CC was inoculated with an equal mixture of strains 204 and A1, both strains exhibited modest but reproducible growth, reaching comparable levels, although strain 204 consistently achieved slightly higher densities (Fig. 6A and 6B). Upon supplementation of CC with D‑serine, a significant increase in total biomass was observed, consistent with results obtained in defined M9 medium (Fig. 6A). However, under these conditions, the dsd⁻ strain A1 failed to grow and was eliminated from the culture (Fig. 6B), suggesting that D‑serine or a derived compound exerts a toxic effect on dsd⁻ strains in this environment. This hypothesis was further supported by competition experiments between the wild-type strain 204 and its Δ*dsd* mutant. Addition of D‑serine enhanced the growth of the wild-type strain but led to the disappearance of the Δ*dsd* mutant in CC (Fig. 6C and 6D). These results indicate that the toxic effect of D‑serine is neutralized when the amino acid is catabolized via the Dsd system. To characterize this effect in more detail, we examined the impact of increasing D‑serine concentrations on the growth of strains 204, 204Δ*dsd*, and A1 cultured individually in CC. As expected, D‑serine supplementation increased the biomass of strain 204 at concentrations above 5 mM (Fig. 7). In contrast, D‑serine inhibited the growth of strain 204Δ*dsd* at concentrations as low as 0.1 mM, demonstrating that D‑serine is strongly inhibitory when not catabolized. Growth inhibition was also observed for strain A1, although only at concentrations exceeding 1 mM. The influence of D‑serine on bacterial growth was also confirmed using other HI dsd^-^ and CD dsd^+^ strains from the collection (Figure S4). Collectively, these data demonstrate that D‑serine affects *E. coli* growth in the intestinal environment in two distinct ways: it serves as a nutrient promoting the proliferation of dsd⁺ strains while exerting a toxic effect on dsd⁻ strains.

### Dsd-dependant utilization of **D**‑serine is critical for competitive fitness in the digestive tract

**Fig. 8:**
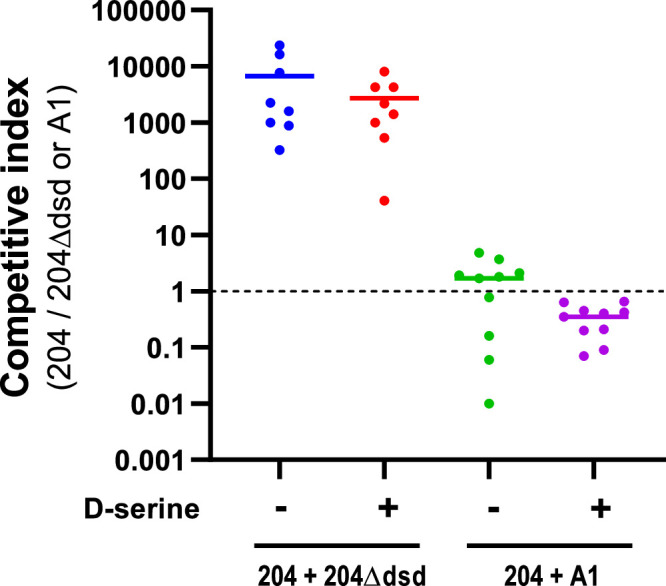
*Role of D-serine and dsd genes in the fitness of E. coli in the mouse gut*. Mice were co-inoculated with an equal mixture of 204 WT and 204∆*dsd* or A1 strains, and D-serine was added to the drinking water when indicated. Five days post-inoculation, feces were sampled and spotted on selective plates and competitive indices (ratio 204 WT/204∆*dsd* or 204/A1) were calculated for each animal.

To assess the role of D‑serine *in vivo*, competition experiments were conducted in the mouse gut. Mice were co-inoculated with equal mixtures of strains 204 and A1, or of wild-type strain 204 and its Δ*dsd* mutant, and bacterial populations were quantified in feces five days post-inoculation (Fig. 8). In mice co-inoculated with wild-type 204 and 204Δ*dsd*, the competitive index reached a mean value of 6 683, indicating that the mutant was strongly outcompeted by the wild-type strain. This result demonstrates that the presence of the *dsd* locus is critical for efficient gut colonization by strain 204.

In contrast, the competitive index obtained from mice co-inoculated with strains 204 and A1 remained close to 1, indicating that both strains exhibited comparable fitness in the mouse gut (Fig. 8). Quantification of L- and D‑serine in cecal contents revealed that L‑serine concentrations were approximately 10- to 50-fold higher than those of D‑serine, with no significant differences between naïve and colonized mice (Figure S3). Attempts to increase intestinal D‑serine levels through supplementation in drinking water did not result in a measurable increase in cecal D‑serine concentrations (Figure S3), and competitive indices remained unchanged (Fig. 8).

## Discussion

The intestinal microbiota of patients suffering from digestive or extra-digestive diseases is frequently altered, and among the various hallmarks of dysbiosis, the expansion of *Enterobacteriaceae* is one of the most commonly observed features. Several hypotheses have been proposed to explain this expansion, including increased oxygen availability favoring facultative anaerobes, as well as inflammatory conditions to which *Enterobacteriaceae* are more resistant than other members of the gut microbiota (Moreira de Gouveia et al., 2024). In the present study, we explored a third hypothesis suggesting that *Enterobacteriaceae* expansion may result from the selection of strains metabolically adapted to the altered gut environment associated with disease.

Using a collection of fecal *E. coli* strains isolated from HI and from CD or RA patients, we systematically compared their ability to catabolize a wide range of carbon substrates, including carbohydrates and amino acids. Only minor differences were observed between isolates from HI and RA patients, as only four carbohydrates were slightly less efficiently consumed by RA strains than by HI strains (Fig. 1A). In contrast, seven substrates were differentially consumed between HI and CD strains, with some differences being particularly pronounced (Fig. 1B). Five substrates were more efficiently utilized by HI strains, whereas two substrates, D-arabinose and D‑serine, were preferentially consumed by CD strains. Overall, the metabolic profiles of strains isolated from CD patients appeared more heterogeneous than those of strains isolated from RA patients. Although both diseases are associated with inflammation, CD is characterized by chronic intestinal inflammation that profoundly alters the local gut environment, which likely explains the greater metabolic variability observed among CD-associated strains.

Among the substrates showing the strongest divergence between HI and CD strains, sucrose and D‑serine emerged as particularly discriminant. Interestingly, the genes involved in the catabolism of these two molecules are located within the same chromosomal region in *E. coli*. The *argW* locus harbors the *dsdCXA* cluster responsible for D‑serine catabolism, but in many strains this cluster is replaced by the *cscRAKB* genes enabling sucrose utilization. The high variability of the *argW* region is attributed to frequent integration events of lysogenic bacteriophages (Moritz and Welch, 2006). In our strain collection, the ability to catabolize D‑serine was approximately twice as frequent among CD strains as among HI or RA strains. This phenotype was strictly associated with the presence of the *dsd* genes, and deletion of the *dsd* cluster in the CD strain 204 completely abolished its ability to utilize D‑serine. In contrast, the association between the presence of *csc* genes and sucrose utilization was less strict (Fig. 3), which may be explained by the existence of alternative sucrose utilization pathways in *E. coli* (Stephens et al., 2024). We further demonstrated that the presence of the *dsd* locus confers a clear fitness advantage to *E. coli* both *in vitro* and *in vivo* (Figs. 5, 6, and 8). This advantage arises through two complementary mechanisms. D‑serine serves as a nutrient that promotes the growth of dsd⁺ strains and exerts a toxic effect on dsd⁻ strains. The bacteriostatic effect of D‑serine has previously been attributed to the inhibition of L‑serine and pantothenate biosynthesis pathways in *E. coli* (Cosloy and McFall, 1973; Maas and Davis, 1950). Although this mechanism was not directly demonstrated in our strains, it likely explains why the A1 and 204∆*dsd* strains did not grow *in vitro* in the presence of D‑serine. Moreover, the toxicity of D‑serine appears to be strain- and environment-dependent. In our study, D‑serine toxicity differed markedly between growth in defined M9 medium and in cecal content medium (Fig. 5, Fig. 6), and also between the dsd⁻ strains A1 and 204Δ*dsd* grown in cecal content (Fig. 7). Similarly, previous studies have reported growth inhibition at 0.5 mM D‑serine for the K-12 strain, whereas no inhibition was observed at 2 mM for the O157:H7 strain EDL933, despite both strains lacking the *dsd* cluster (Connolly et al., 2015; Moritz and Welch, 2006). Beyond its toxic effects, D‑serine has also been shown to act as a signaling molecule modulating gene expression in *E. coli* (Connolly & Roe, 2016). Quantification of D‑serine in mouse cecal contents revealed concentrations of approximately 5 nmol/g, consistent with micromolar levels previously reported in the literature (Bastings et al., 2019; Connolly et al., 2015), but substantially lower than the millimolar concentrations required to observe growth effects *in vitro*. Nevertheless, deletion of the *dsd* locus in strain 204 resulted in a dramatic fitness defect in the mouse gut, with the mutant being outcompeted by the wild-type strain by approximately four orders of magnitude (Fig. 8). These results indicate that D‑serine catabolism plays a critical role in *E. coli* fitness within the gastrointestinal tract. This major fitness defect of the mutant occurs despite the fact that D‑serine was detected at micromolar concentrations *in vivo*. This discrepancy may be explained by several hypotheses. First, D‑serine was quantified from bulk cecal contents, and we cannot exclude that its availability may be higher in other gut compartments or even locally within specific microenvironments. As mentioned above, D‑serine is known to interfere with functions other than metabolism in *E. coli*, through toxic effects on certain reactions or through regulatory effects on gene expression. Such pleiotropic effects might explain why deletion of the *dsd* locus has a profound impact on the fitness of *E. coli* strain 204 in the gut, even at low concentrations of D‑serine. We also attempted to artificially increase D‑serine levels in the mouse cecum through supplementation in drinking water but this approach was unsuccessful. This suggests that D‑serine is likely absorbed, metabolized, or degraded in the upper intestinal tract. Unexpectedly, the CD dsd^+^ strain 204 and the HI dsd^-^ strain A1 exhibited comparable fitness during gut colonization, despite their opposite capacities to utilize D‑serine. One limitation of our model is the pretreatment of mice with streptomycin. Although this treatment facilitates the establishment of inoculated strains, it strongly alters the gut microbiota and simplifies ecological competition. Moreover, our model does not account for the intestinal inflammation observed in CD patients. Alternatively, the comparable fitness of strains 204 and A1 in our model might be explained by other strain-specific traits that compensate for the lack of D‑serine catabolism in strain A1. Phenotype microarray data indicate for example that strain A1 more efficiently utilizes several other substrates, including L-glutamine, sucrose, β-methyl-D-glucoside, and D-malic acid.

The enhanced ability of CD strains to utilize D‑serine is consistent with metabolomic studies reporting increased concentrations of free amino acids in the feces of pediatric CD patients (Ni et al., 2017). In CD patients, the release of ammonia from host urea hydrolysis promotes the synthesis of amino acids by the gut microbiota, and elevated amino acid levels have been positively correlated with disease activity and with increased abundance of Proteobacteria, including *E. coli* (Ni et al., 2017). In line with these findings, it has been shown that adherent-invasive *E. coli* rely on amino acids, particularly L‑serine, to gain a fitness advantage in the inflamed gut of CD patients (Kitamoto et al., 2020). The importance of amino acid utilization in other intestinal disease contexts has also been demonstrated for *Clostridioides difficile* and colibactin-producing *E. coli* (Battaglioli et al., 2018; Devaux et al., 2025).

Together, these observations support the notion that free amino acids, which are abundant in inflamed intestinal environments, constitute a pool of substrates exploited by *E. coli* and other members of the Proteobacteria to proliferate during dysbiosis. While most amino acid catabolic pathways belong to the *E. coli* core genome, some pathways, such as the *dsdCXA* cluster examined in this study, are part of the accessory genome. The uneven distribution of such genetic traits shapes the metabolic potential of individual strains and may influence their fitness within specific environments. Consequently, alterations of the gut metabolic landscape, as observed during intestinal inflammation, are likely to promote the selection and expansion of metabolically adapted strains. Further investigations are required to demonstrate that D‑serine consumption enhances *E. coli* proliferation in the gut of CD patients and, more generally, to better understand the contributions of L- and D-amino acids and their dedicated catabolic pathways to *Enterobacteriaceae* colonization and expansion in dysbiotic conditions. Such studies may ultimately pave the way for novel therapeutic strategies aimed at limiting the overgrowth of *E. coli* and other Proteobacteria in patients suffering from diseases associated with intestinal dysbiosis.

## Declaration of competing interest

The authors declare that they have no known competing financial interests or personal relationships that could have appeared to influence the work reported in this paper.

## Appendix Supplementary materials

Image, application 1
