# Supplementary material for: D-serine metabolism enhances Escherichia coli fitness in the gut and could contribute to Enterobacteriaceae expansion in Crohn's disease patients
Source: Curr Res Microb Sci. 2026 Mar 23;10:100585. doi: 10.1016/j.crmicr.2026.100585 (PMC13054288; doi:10.1016/j.crmicr.2026.100585)
Supplement: Supplementary file 1 [file mmc1.docx]

**SUPPLEMENTARY DATA**


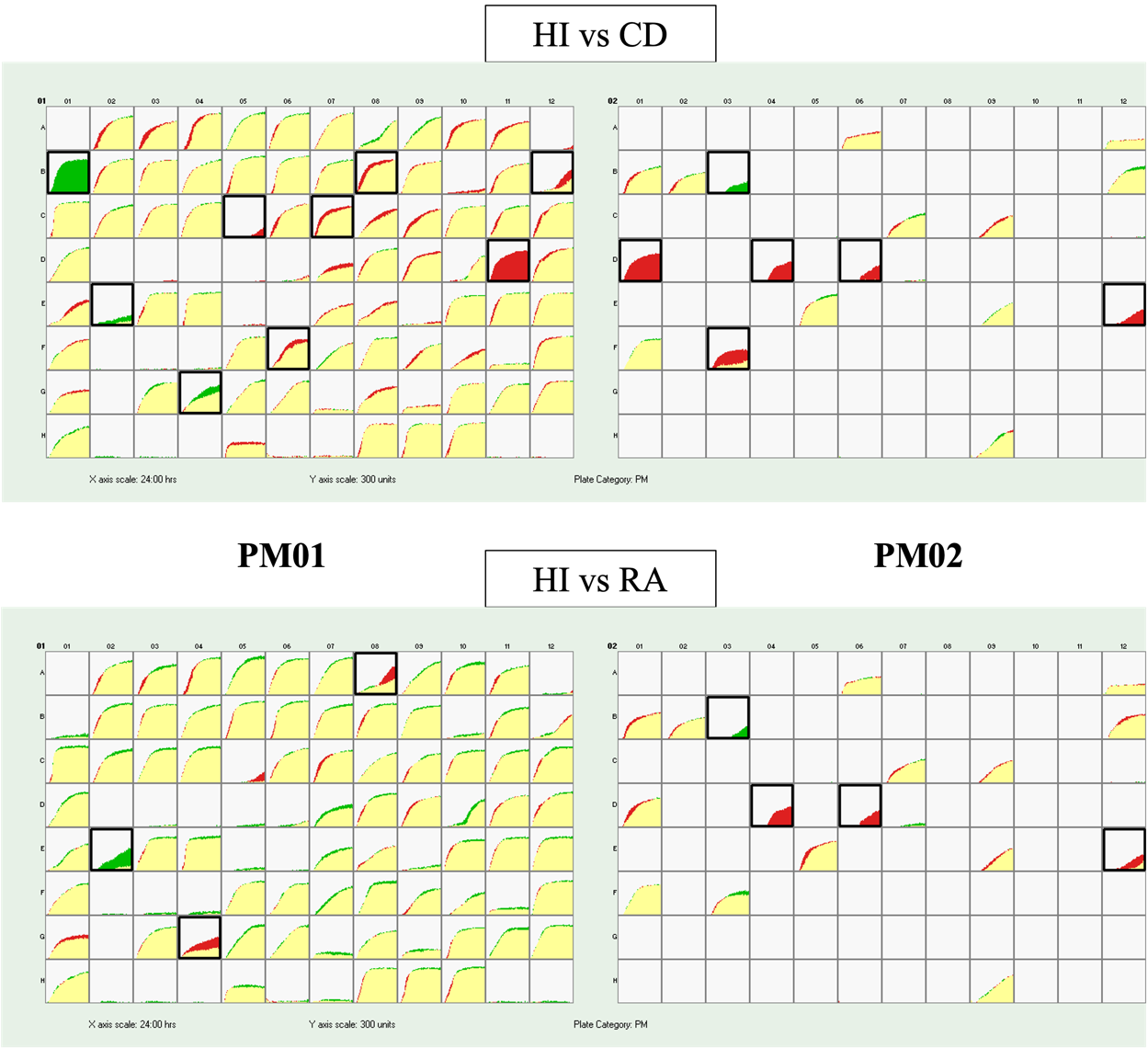


**Figure S1: Growth comparisons between HI, RA and CD strain groups for 190 carbon substrates.** Plates PM01 and PM02 were individually inoculated with 16 HI strains, 14 RA strains or 15 CD strains. After a 24 h period incubation, means of AUC were represented for each well as follow: yellow colored AUC indicate similar growth between HI and CD or RA strain groups; green colored AUC indicate a higher growth for CD or RA strain group than HI strain group; red colored AUC indicate a higher growth for HI strain group than for CD or RA strain group.


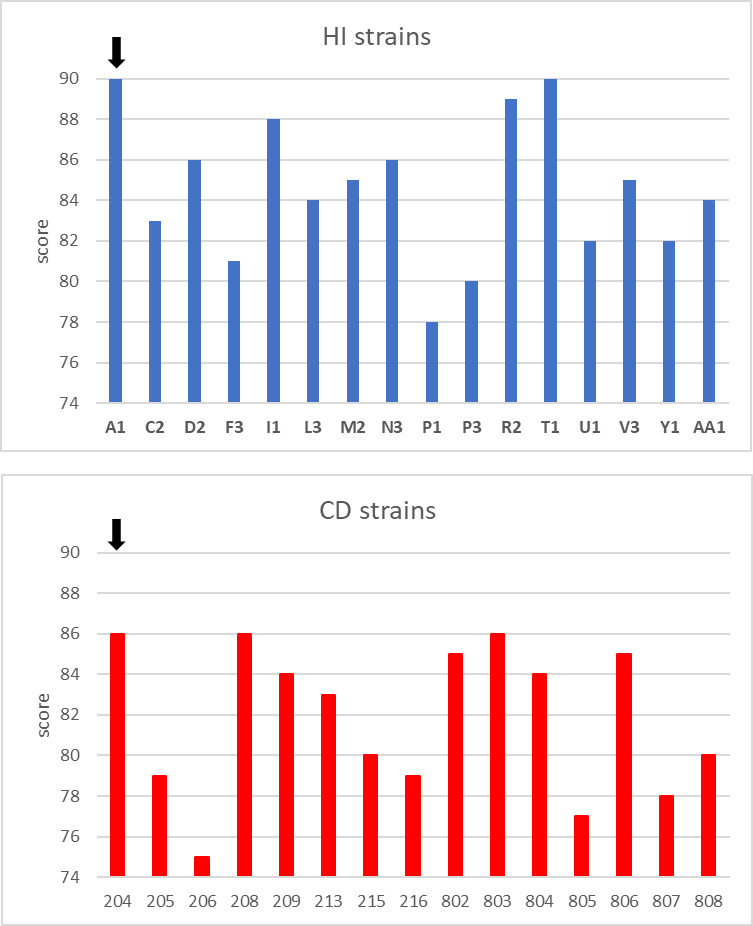


**Figure S2: Determination of the best representative strains for HI and CD groups.** A score was attributed to each strain based on adequacy between substrate utilization of individual strains and average utilization by their corresponding strain group (see M&M for details). Black arrows indicate representative strains selected to represent the HI or CD strain groups.

**Figure S3: D-serine quantification from *in vitro* and *ex vivo* samples and D- and L-serine quantification from *in vivo* samples.** Each dot represents a replicate and lines show the mean values. A two-way ANOVA with Holm-Sidak's correction was performed to compare groups.

**Figure S4: Effect of D-serine on the growth of additional HI and CD strains.** Growth was performed in mouse cecal contents supplemented or not with 20 mM D-serine. OD_600nm_ was measured at 8h and values are shown as percentages relative to OD_600nm_ measured without D-serine supplementation for each strain. Data represent the mean and standard deviation from 3 independent replicates.

**Table S1. Bacterial strains and plasmids used in this study**

| Strains or plasmids | Description | References |
| --- | --- | --- |
| *Bacterial strains (phylogroup)* |  |  |
| A1*(D)*, C2*(B2)*, D2*(B2)*, F3*(B2)*, I1*(D)*, L3*(B1)*, M2*(B2)*, N3*(B1)*, P1*(B2)*, P3*(B1)*, R2*(D)*, T1*(D)*, U1*(F)*, V3*(A)*, Y1*(D)*, AA1*(D)* | E*. coli* strains isolated from fecal samples of healthy individuals | (Moreira de Gouveia et al., 2022) |
| 204*(A)*, 205*(B2)*, 206*(A)*, 208*(B2)*, 209*(C)*, 213*(B2)*, 215*(C)*, 216*(D)*, 802*(C)*, 803*(F)*, 804*(B1)*, 805*(A)*, 806*(A)*, 807, 808 | *E. coli* commensal strain isolated from patients suffering with Crohn’s disease | This study |
| W1*(A)*, W4*(A)*, W5*(B1)*, X4*(B2)*, AQ1*(D)*, AQ3*(B2)*, AR1*(B1)*, AU1*(B2)*, AS2*(A)*, AV1*(B2)*, AW1, AW2*(A)*, AW3*(C)*, AX1 | *E. coli* commensal strain isolated from patients suffering with rheumatoid arthritis | This study |
| 204-Gm | 204 strain with an insertion of a Gm^R^ cassette at the *att*Tn7 site, Gm^R^ |  |
| 204 Δ*dsd* | 204 strain with a deletion of genes *dsdC*, *dsdX* and *dsdA*, Kan^R^ | This study |
|  |  |  |
| *Plasmids* |  |  |
| pKD4 | *kan* cassette template, Amp^R^, Kan^R^ | (Datsenko and Wanner, 2000) |
| pGP-Tn7-Gm | *oriR6K mobRP4 Tn7-Gm; Ap^R^ Gm^R^* | (Crepin et al., 2012) |
| pSTNSK-Km | *oriSC101*(Ts) *tnsABCD*; Km^R^ | (Crepin et al., 2012) |

**Table S2. Primers used in this study**

| **Purpose** | **Name** | **Sequence 5’-3’** | **References** |
| --- | --- | --- | --- |
| 16S | 8F | AGAGTTTGATCCTGGCTCAG | (Edwards et al., 1989) |
|  | 1492R | CGGTTACCTTGTTACGACTT | (Lane D.J., 1991) |
| RAPD | 1283 | GCGATCCCCA | (Nielsen et al., 2014) |
| Detection of *dsd* and *csc* locus | dsdA_R | CGGATACTGGGCGATGAGCG | this study |
|  | cscR_F | GGCGTGCGACATCCTTTAATG | this study |
|  | dsdX_F | CTACCGTCATTAGGCGCAACG | this study |
| 204 ∆*dsd* mutant |  |  |  |
|  | dsdmut_F | CTGCCTGCCCGGTAATGTGGTGATGTAATAATGCTGATGGCATTTCACCGGTGTAGGCTGGAGCTGCTTC | this study |
|  | dsdmut_R | CGGCACCATTCCACCTCCCGTCGCCCACACCAGATGAGTGGCATTACGCACATATGAATATCCTCCTTAGTTCC | this study |
|  | yfdC_F | ACGACATGAGCAACAAGCGC | this study |
|  | emrY_R | GTTTACAGCGAAAGGCGTTGGG | this study |

**References**

Crepin, S., Harel, J., Dozois, C.M., 2012. Chromosomal complementation using Tn7 transposon vectors in *Enterobacteriaceae*. Appl. Environ. Microbiol. 78. https://doi.org/10.1128/AEM.00986-12

Datsenko, K.A., Wanner, B.L., 2000. One-step inactivation of chromosomal genes in *Escherichia coli* K-12 using PCR products. Proc. Natl. Acad. Sci. U. S. A. 97, 6640–6645. https://doi.org/10.1073/pnas.120163297

Edwards, U., Rogall, T., Blöcker, H., Emde, M., Böttger, E.C., 1989. Isolation and direct complete nucleotide determination of entire genes. Characterization of a gene coding for 16S ribosomal RNA. Nucleic Acids Res. 17. https://doi.org/10.1093/nar/17.19.7843

Lane D.J., 1991. 16S/23S rRNA Sequencing, in: Nucleic Acid Techniques in Bacterial Systematics. pp. 115–148.

Moreira de Gouveia, M.I., Daniel, J., Garrivier, A., Bernalier-Donadille, A., Jubelin, G., 2022. Diversity of ethanolamine utilization by human commensal *Escherichia coli*. Res. Microbiol. 103989. https://doi.org/10.1016/j.resmic.2022.103989

Nielsen, K.L., Godfrey, P.A., Stegger, M., Andersen, P.S., Feldgarden, M., Frimodt-Møller, N., 2014. Selection of unique *Escherichia coli* clones by random amplified polymorphic DNA (RAPD): Evaluation by whole genome sequencing. J. Microbiol. Methods 103. https://doi.org/10.1016/j.mimet.2014.05.018
